# Host Country Regulation of Short-Term Medical Missions: Evidence from Three Countries

**DOI:** 10.5334/aogh.4896

**Published:** 2025-11-10

**Authors:** Erwin Calgua-Guerra, Efua Esaaba Mantey, Emilly Comfort Maractho, Daniel Doh, Guillermo Zea-Flores, Carolina Lopez, Sirry Alang, Peter Donkor, Virginia Rowthorn, Judith N. Lasker

**Affiliations:** 1 School of Medicine, University of San Carlos of Guatemala, Guatemala City, Guatemala; 2 Department of Social Work, University of Ghana, Legon, Accra, Ghana; 3 Uganda Christian University, Mukono, Uganda; 4 School of Allied Health, University of Western Australia, Perth, Australia; 5 Rafael Landívar University, Guatemala; 6 Independent Researcher, Guatemala; 7 University of Pittsburgh, USA; 8 Professor Kwame Nkrumah University of Science and Technology, Kumasi, Ghana; 9 University of Maryland, Baltimore, MA, USA; 10 Lehigh University, Bethlehem, PA, USA

**Keywords:** short-term medical missions, regulations, ghana, uganda, guatemala

## Abstract

*Background:* Globally there is a grown concern about the ethics and effectiveness of short-term global health activities, also known as short-term medical missions (STMMs). Guidelines for better practices have been developed exclusively by organizations in the Global North, with no mechanisms to enforce and monitor any of these.

*Objective:* The goal of this study is to analyze regulations in three host countries where such STMMs take place.

*Methods:* Researchers from Ghana, Uganda, and Guatemala were recruited to investigate regulations affecting STMMs. Interviews were performed with 129 participants, including policymakers, health services staff and administrators, patients, and community leaders. Data was analyzed using NVIVO and R Package for Qualitative Analysis, having as the foundation grounded theory and sentiment analysis for identification of patterns in responses.

*Findings:* In all three countries, there are regulations regarding the practice of health care generally, as well as governing the importation of medications, but these are rarely enforced when it comes to STMMs, and many participants were unaware of them. In Ghana and Uganda, there is no specific regulation for STMMs. In Guatemala, specific and detailed regulations do exist governing the practice of STMMs, but participants did not report awareness of these regulations.

*Conclusions:* The lack of explicit regulation of STMMs in some countries and the lack of enforcement of existing professional regulations and rules on importation of medications can easily be exploited by those volunteers who prefer not to follow them. All three countries studied do have procedures to be followed by any person who intends to perform STMMs.

## Introduction

Short-term experiences in global health (STEGH), also known as short-term medical missions (STMMs), usually last in the range of one to two weeks and focus on health problems that institutions, professionals, and volunteers from high income countries (HICs) perceived as facing low- and middle-income countries (LMICs) [1].

Despite the good will that motivates such efforts, there has been growing attention to the shortcomings of STMMs. Therefore, there has also been a proliferation of guidelines for better practices, developed almost exclusively by scholars and professional organizations in the Global North [2–9]; unfortunately, there are essentially no mechanisms to enforce any of these.

Rowthorn et al. [10] have reviewed the legal aspects of these programs and point out that “not every STEGH organization is aware of, or adheres to, these guidelines, and some organizations actively or tacitly allow unethical and potentially illegal practices. Certain US-based organizations brazenly market STEGH programs in LMICs as a means to circumvent regulations at home.” Two key issues addressed in Rowthorn’s analysis are the practice of medicine by untrained volunteers and the importation of medications without appropriate controls for quality and adherence to local laws and regulations.

Lacking authority and jurisdiction, the regulatory agencies of HICs, where most volunteer programs originate, rarely exercise any control over the quality and appropriateness of STMMs outside of their borders. Doing so is left up to sponsoring organizations which have many other priorities in planning their programs [11].

Regulations governing practice by medical professionals exist in most countries in the world. In recent years, there has also been a marked increase in host country regulations around importation of donated supplies and medications [12]. Yet in most countries where STMMs take place, regulatory agencies are under-resourced and lack the ability to engage in meaningful enforcement.

The goal of this paper is to consider regulations regarding STMMs in three countries that are frequent hosts of visiting teams—Ghana, Uganda, and Guatemala; this is a topic on which, to our knowledge, there is almost no research.

## Methods

Local researchers were recruited to investigate what laws and policies exist in the host countries to govern the practices of STMMs and to what extent they are effective in doing so.

All the studies were approved by the Lehigh University Institutional Review Board (1223538) and by a review committee in the relevant country (Ghana Health Service Research Ethic Board GHS-ERC005/03/18, Makerere University School of Social Sciences Research Ethics Committee MAKSS REC 09.18.216, and ZUIGEME PROZU514-19, in Guatemala).

Information about the legal and policy context was gathered in all three countries by a review of regulatory documents and interviews with administrative officials. Additionally, all research teams interviewed medical and administrative staff who have experience with hosting STMMs. Finally, media reports regarding cases brought by professional medical councils were reviewed.

In Ghana, researchers carried out in-depth interviews with 24 respondents who were purposefully selected from health-care institutions, public and private, in several regions, based on consultation with Ghanaian experts in public health regarding areas with a high concentration of STMM activities. There were seven hospital administrators, two officials of professional regulatory agencies, eight physicians, and seven nurses, and they were all asked about regulations on STMMs [13].

In Uganda, a total of 46 key Informant Interviews were conducted in person by Ugandan researchers in 10 locations, based on perceived high level of engagement with STMMs and ensuring rural and urban locations [14].

The 46 participants included 17 policymakers (Ministry of Health, District Health Services, Chief Administrators at Local Government, and Private Health Providers), 19 administrators and staff of host institutions (NGOs and media), and 10 host community members (Local Council representatives at village level and patients who were participants in an STMM).

In Guatemala, investigators identified key informants from the Ministry of Public Health and Social Assistance (MSPAS) and the College of Surgeons of Guatemala (COLMED). An Internet search led to documents and regulations on STMMs in Guatemala.

Based on the list of upcoming STMMs provided by COLMED, two organizations located in the rural area and two in the urban area were randomly selected and contacted for the study. Additionally, a program that had not registered with COLMED was added to the interview schedule. It is important to note that due to the COVID-19 pandemic, interviews and focus groups could be conducted at only three of the planned locations [15].

A total of 40 individual interviews and 6 focus groups were conducted in three locations. These were all nongovernmental hospitals founded by humanitarian and faith-based organizations.

Of the interviews conducted, eight were with health-care staff and administrators and 32 were with patients.

## Results

### International law and policy

International institutions exist to assess, enhance, and guide health provision. The World Health Organization (WHO) is of course the most important in setting goals and policies. As Rowthorn et al. observe that

WHO is the global leader in setting standards, underpinned by science, ethics, and human rights, expected by the world community when engaging in health-related activities. Thus, while these guidelines do not have the force of law, they are the written embodiment of international standards developed through a consensus-building process. Hence, STEGH organizations that do not follow the guidelines are essentially acting in a rogue manner vis-à-vis the community of nations [10].

An example of the potential role of international law and agreements can be seen in the case of donated medications:

The use of donated drugs for humanitarian purposes is specifically discouraged by WHO and Federal Drug Administration (FDA). In 2010, WHO published “Guidelines for Medicine Donations.” The document specifically covers drug distribution by private volunteer organizations and other groups that could be considered STEGH organizations, and provides examples of poor medicine donation practices [10].

### National policies regarding STMMs

Rowthorn and colleagues found that “virtually all countries have a medical licensure framework” that requires licensing from the government to practice medicine in that country. Most of these have a provision for short-term licensing of visiting practitioners, generally by application and payment of a fee. We first review the laws in the three countries under study.

#### Ghana

Many different governmental and organizational actors are potentially involved in regulating STMMs. Some provide regulatory and oversight roles through registration and licensing of health professionals. Others provide the policy context and health infrastructure for STMM activities. Even the Ministry of Foreign Affairs contributes by facilitating visas.

The Ghana Health Service’s Code of Conduct makes it mandatory for all health professionals to be registered and to remain registered with their professional regulatory bodies. Subsequently, the Health Professions Regulatory Bodies Act, which was issued in 2013, set up the Allied Health Professions Council, the Council, the Nursing and Midwifery Council, the Pharmacy Council, and the Psychology Council, “to secure in the public interest the highest standards of training and practice,” and with the mandate to ensure standards, training, registration, and regulation of all these health-related professionals. There are no regulations specifically designed for STMMs; however, the Councils have the ability and authority to provide temporary licenses to health professionals who wish to practice in Ghana for three months or less [16].

Respondents from Ghana’s regulatory institutions indicated that, because STMMs are providing health care in Ghana, they are subject to national laws and policies governing such care. For example, temporary registrants practice only in an “approved or accredited health facility,” which would preclude pop-up clinics that typify many STMMs. Temporary registration requires evidence of appropriate degree and licensing, letters of reference, an invitation from the Regional Director of Health Services in the region of Ghana where the person will be working, and payment of a fee. Up to this date, there is little evidence of routine control and coordination of volunteers who practice in the country.

In 2019, the FDA in Ghana issued guidelines for donations of medications. These include documenting a need for the specific donations and that the donations are consistent with products already approved and in use in the country. It should be clear who is responsible for receipt of the drugs and also that adverse effects are reported. This request must be made at least one month before importation, and there are fines and imprisonment defined for those who do not follow the rules [17].

#### Uganda

Uganda also has rules regarding registration and licensure of health-care practitioners. As is the case in Ghana, there are no health regulations specifically tailored for STMMs. As in Ghana, there is a clear set of procedures for applying for temporary registration, including provision of proof of medical qualifications and licensing, a letter of invitation from a Ugandan institution and letters of reference. Also required is clearance by Interpol and the payment of an application fee [18].

The Ugandan health-care sector is regulated by different frameworks that operate at different governmental levels, with the Health Sector Development Plan being the most comprehensive of all. The Local Government Act provides decentralizing health-care provision to districts. There are separate bodies of law that regulate specific aspects of health-care provision, such as HIV/AIDS, registration of medications, importation of medications, among others.

The Uganda Medical and Dental Practitioners Act of 1998 established the Uganda Medical and Dental Practitioners’ Council, whose responsibilities include monitoring the quality of training and qualifications of members of the health service and enforcement of professional ethics. The act stipulates that only registered practitioners can practice in the country.

*The National Drug Policy and Authority (NDA) (Importation and Exportation of Drugs) Regulations, 2014* Regulation 3(1), require a license for any drugs entering Uganda. In 2018, the NDA issued a reminder to the public of procedures governing the importation of donated drugs specifically. These include the recipient institution documenting the need for the products and their appropriateness for the population, inspection and verification by an NDA official upon arrival, and (with the exception of vaccines) they should have a remaining shelf life of at least one year [19].

#### Guatemala

COLMED requires foreign professionals to “submit in writing to the College of Physicians and Surgeons of Guatemala... the authorization request form for the temporary professional practice of professionals, at least fifteen business days in advance...” [20]. Regulations define the documentation to be submitted by each professional, and COLMED must determine if the professionals are duly licensed in their home countries.

Also, similarly to the other two countries, Guatemala requires that all pharmaceuticals and medical supplies be registered with the Department of Regulation and Control of Pharmaceuticals and Related Products. “Importing donated products necessitates a comprehensive set of documents, including payment receipts, invoices or product lists with detailed information, and specific forms signed by the legal representative of the receiving entity” [21].

In contrast to Ghana and Uganda, Guatemala *does* have regulations that apply specifically to the practices of STMMs, which in Guatemala are called “jornadas”—medical days” or “health days.” The Ministry of Public Health and Social Assistance (MSPAS) established mandatory requirements for jornadas in a Technical Standard 35-2019, “Authorization of Medical or Health Days”, that assigns responsibility for oversight of all type of jornadas to the Department of Regulation and Accreditation and Control of Health Establishments (DRACES) [22].

All jornadas anywhere in the country must submit documentation to DRACES, which issues a letter authorizing the program if it meets the criteria established, beginning with a responsible licensed health professional authorized to practice in Guatemala. Municipal Health Districts have the responsibility for direct oversight of the physical conditions, materials, and execution of the programs and must issue a document to that effect. At hospitals, the General Director is responsible for documentation as well as supervision. Letters verifying the proper conditions and requesting authorization to proceed must be submitted to DRACES either by the Municipal Health District or the General Director of the hospital at least eight business days in advance of the jornada.

Additionally, there must be a letter of commitment from the person directly responsible for the jornada. The letter must include the names and credentials of all who will participate, with proof of fitness from the National Registry of Sexual Offenders. The responsible person also must submit records of patients treated and referred elsewhere to the Health District.

The standard also identifies specific prohibitions, such as performing against laboratory tests in unauthorized facilities or providing treatments that require highly specialized professionals who are not available.

Another requirement is that the STMM should be free or very low cost and of interest and benefit to the population. In addition, it is clarified that STMMs are not “practice or training” activities for resident doctors, so such doctors can only be allowed to assist in surgical procedures as long as a qualified surgeon is present to supervise. Medical professionals and nurses must have a minimum of five years of professional practice in their respective countries. Another important aspect is that the parties involved should establish agreements or letters of understanding.

### Enforcement and awareness of regulations

Internationally adopted policies and regulations have no enforcement mechanisms. Adhering to these standards relies on voluntary action on the part of the individual countries.

Rowthorn and colleagues observe that

Many STEGH organizations and participants do not inquire about, nor seek, temporary licensure or appropriate training affiliation agreements in host countries. Licensed US health care providers often assume, or are told by the US-sponsoring organization, that their US licensure is sufficient. When inquiries are made, many are told that the sponsoring organization or local clinical site is “taking care of it” or that it is too administratively burdensome to get a license, and that engaging in that process will take medical care away from those who need it. To the extent that an individual practices medicine without a license from, or the specific authorization of, the host country’s licensing body, the individual is likely violating the law of the host country, even if the unlicensed activities appear to be tacitly endorsed in a particular clinical setting.

#### Ghana

All participants were asked about their knowledge of laws or policies that regulate the activities of STMMs. Apart from the 2 respondents from regulatory institutions, none of the 20 other participants, who were health professionals and hospital administrators, had any knowledge of a legal or policy framework that would apply to STMMs.

As some interviewees noted, it is up to the host organizations rather than the professional bodies or the Ministry of Health to enforce the rules for the benefit of their communities. Even a representative of one of the regulator councils agreed about this:

*I think the problem with our volunteers is that they are scattered, not well coordinated from above, so everyone is doing his own thing in his own way but there should be a coordinated unit, may be in the Ministry of Health (MoH) or the Ghana Health Service…* (medical officer/surgeon).

While most respondents indicated that they were not aware of specific laws or policies for STMMs, some were still confident that professional regulation of some kind existed and that others were paying attention to them.

Official of rural NGO: *I think the Social welfare may have laws like that. But I do not know how those laws apply. I think there needs to be more education on that.*

Physician: *I do not know of any law. What I know is that they deal with the administration directly.*

Hospital administrator: *I am sure they have a protocol and that would include verifying the competence or even the qualification of those people as to whether they are qualified to practice medicine before they would be allowed.*

And a few were quite specific in how they do adhere to the regulations:

Hospital administrator: *They cannot just go and put up a tent and say everybody should come to have access and so on. The laws of Ghana would not allow that. So, when you go you need to attach yourself to a health care institution. We go to the Medical and Dental Council to get a temporal license for all the doctors who are coming and to the Nurses and Midwifery Council to get a temporary license for them so that they can honor our patients.*

Medical officer in a municipal hospital: *Sometimes some volunteers came in parading as specialists, and they turned out to be students. Once you find that out, you need to be able to stop them. I know the Medical and Dental Council is being blamed for being hostile to people outside, but they do this because they need to protect the public and guide the profession.*

#### Uganda

Most of the participants had limited or no awareness of the laws and policies. Of 17 policymakers and NGO staff and 19 program organizers, only 9 (25 per cent) knew that there are regulations, and these were all in the category of policymakers. Even among those who said they were aware that policies and laws existed, the majority (6/9) could not name anything specific.

Interviews revealed awareness on the part of some participants regarding the requirement of registering with the appropriate professional council.

District Health Officer: *You must get a work permit before you work here. They acquire visas through the institutions they are coming to visit…You cannot just come as a foreigner and begin bragging and begin working as a medical officer or nurse without the relevant council registering you.*

Hospital Medical Director: *We’ve always encouraged them to register with the Uganda Medical and Dental Practitioners’ Council, so they usually apply to the council and then present their papers, are vetted and then allowed to come and practice.*

Notable in this last quote is that the hospital “encourages” visitors to register, and they “usually” do so. Others also referred to these practices “usually” being followed.

It is also difficult to verify how many STMM personnel submit their imported medications to the NDA for inspection. Observations by informants going through the airport suggest that there may be more vigilance in publicizing the regulations and checking baggage than in the past.

But there was also expression of the need for a more appropriate legal and policy framework for the programs a whole and to keep people from avoiding laws.

One policymaker in health made the point that

*There is need for reforms in policy; government should look at their period of stay. The volunteers should be scrutinized and sent to areas of operation according to their areas of expertise and needs of particular areas*.

#### Guatemala

Despite the existence of regulations specific to STMMs, neither COLMED nor MSPAS appear to have the resources to ensure that those regulations are being followed. There are some organizations carrying out STMMs without requesting authorization to do so. The researchers were struck by the ease with which permission can be obtained. At COLMED, only a list of those who make up the group is required, as well as a copy of the professional title of the person who heads it and the name, membership number, and seal of the licensed professional responsible. Of greatest significance, DRACES lacks the staff and financing to provide any enforcement of this standard in addition to all their other responsibilities.

Of the eight health facility staff and regulators interviewed, all were aware of the presence of laws and regulations governing STMMs. As one reported that

*There are laws that apply to them, complying with all the provisions put to us by the Medical College. All the laws are complied with, because they also conform to the hospital’s standards. Before you can plan a jornada, you make an agreement or a contract with the different institutions that come.*

It is important to note that all interviewed staff were based in hospital settings with regular visiting teams and that these teams had all registered their visits with COLMED. One reported that “*everyone who comes is already certified by the Medical College to be able to work here, because the medical college demands all that; more than a month or a month and a half before, they send their entire file.*”

Some Guatemalan hosts perceived the rules as onerous. One respondent noted that *The country’s bureaucracy has many requirements to be able to help people and delay things that are already established.*

## Discussion and Conclusions

First, we note that there are other legal issues that require attention. One is the question of accountability in the case of malpractice. Although there may be laws allowing for a civil suit to be instituted against foreigners, it is a long, time-consuming procedure with costs that patients may not be able to afford. Not fearing consequences for errors or complications surely reinforces the likelihood of visitors to ignore other requirements.

In three different nations, we found that there are laws and regulations regarding the practice of medical, dental, and nursing care generally, but that these are rarely or at least not uniformly enforced when it comes to STMMs. In Ghana and Uganda, there is no specific provision for the regulation of STMM *programs*, although there are laws and policies regarding professional licensing of anyone practicing in the country as well as control over donated medications and equipment, all of which do apply to STMMs. In Guatemala, specific regulations do exist for obtaining approval both for an STMM program and for certifying qualified practitioners and medications, but participants did not report awareness of these regulations and the office designated to enforce the program regulations does not have the funding and staff to do so.

The majority of host health workers interviewed in Ghana and Uganda were unaware of what regulations might apply to STMMs. In contrast, Guatemala’s regulations around STMM programs were known to study participants, who regularly host teams in their hospitals.

In addition, these medical missions fall between the cracks of multiple legal frameworks—Ministry of Health, Immigration Service, and civil courts. These three countries have very robust legal and regulatory frameworks when it comes to immigration, medical licensure, and pharmaceuticals, but no single agency clearly has the authority to enforce these various laws in the case of STMMs. The most relevant is the Ministry of Health and the health licensing (registration) boards for each health professional groups, but they are very understaffed. In Guatemala, DRACES in MSPAS is specifically charged with oversight of STMM activities, but we were not able to obtain any information from this office about incoming medical teams.

It is important to acknowledge that many of these regulations are relatively recent in passage.

There are a number of additional explanations related to the history and political economy of the STMM phenomenon as well as other forms of “humanitarian” or “development” endeavors. According to Rowthorn, “Unethical and illegal activities by some STEGH organizations may exist because of an outdated charity model of aid that ignores the complex long-term needs of LMIC health care systems, populations, and laws…Some organizations may also believe that bypassing burdensome legal constraints is a necessary short-cut to meet the needs of underserved communities.”

Prior research on the failure of sending organizations in the United States to comply with ethical principles and standards identified competing priorities that reduce the likelihood of such compliance [11]. Organizations are often focused on the role of STMMs in enhancing their reputations and financial well-being as well as recruitment and retention of employees or students. Achieving these goals may conflict with a focus on ethical and legal standards. Additionally, the logistical arrangements involved in planning a trip are often very demanding, leaving little time and energy for following rules that may be unknown or unenforced.

Nevertheless, it is the visitors’ duty to inquire widely as to the applicable laws, and some medical mission/STEGH groups do a good job with learning and following the regulations. As Lawrence Gostin writes [23], “It is so obvious that having to say it highlights a disturbing complacency, ignorance, or simple arrogance on the part of some STEGH organizations and participants. Just as the United States has robust licensure and practice regulations, so do host countries. Although visitors may be unaware of these laws, or find them administratively burdensome, these same considerations would never be an excuse to flout the law at home. And the fact that some volunteers perceive they can get away with it because LMICs supposedly don’t systematically enforce these laws is a shocking lack of respect for the host country and its population.”

There are also competing interests in the host countries. Host officials may consider the medical and economic benefits to themselves and their communities as valuable enough so as not to impose constraints on the visitors despite the fact that an estimated 18% of the total cost of the STMMs goes into the community [3]. Political figures may see the facilitation of visiting teams a sign of their value to their communities even if it means circumventing the rules. Everyone involved finds it easier—less time-consuming and less costly (fees can be hundreds of dollars for each visitor to be registered)—to ignore or work around the regulations.

The lack of explicit regulation of STMMs in some countries can easily be exploited by those who prefer not to go to the trouble of following the laws that do apply. Registration of STMM volunteers is a function that governments should embrace and treat with utmost importance; otherwise, the lives of sick people are exposed to unqualified and unregulated volunteers and students.

Currently, there is limited choice for those host communities. They receive whoever is interested in working with them and usually appreciate the offers of help, and many have limited awareness of the laws and policies.

Requirements for all medical practitioners in the country to be approved by the pertinent professional boards could and, in our view, must be applied to all visiting short-term medical professionals in order for them to participate in STMMs. This will require better communication of the rules to host organizations as well as to sending organizations and the imposition of fines or other consequences for failure to comply. It will also require more careful oversight of visiting teams.

As long as there are no consequences to the outside medical teams for failing to comply with registration and certification requirements, the incentives to avoid them will continue to prevail.
